# Therapeutic mechanism of baicalein in peritoneal dialysis-associated peritoneal fibrosis based on network pharmacology and experimental validation

**DOI:** 10.3389/fphar.2023.1153503

**Published:** 2023-05-17

**Authors:** Xiaohui Lu, Kefei Wu, Simin Jiang, Yi Li, Yating Wang, Hongyu Li, Guanglan Li, Qinghua Liu, Yi Zhou, Wei Chen, Haiping Mao

**Affiliations:** ^1^ Department of Nephrology, The First Affiliated Hospital, Sun Yat-sen University, Guangzhou, China; ^2^ NHC Key Laboratory of Clinical Nephrology, Guangdong Provincial Key Laboratory of Nephrology, Sun Yat-sen University, Guangzhou, China

**Keywords:** chronic kidney disease, peritoneal dialysis, peritoneal fibrosis, encapsulating peritoneal sclerosis, baicalein

## Abstract

Baicalein (5,6,7-trihydroxyflavone) is a traditional Chinese medicine with multiple pharmacological and biological activities including anti-inflammatory and anti-fibrotic effects. However, whether baicalein has a therapeutic impact on peritoneal fibrosis has not been reported yet. In the present study, network pharmacology and molecular docking approaches were performed to evaluate the role and the potential mechanisms of baicalein in attenuating peritoneal dialysis-associated peritoneal fibrosis. The results were validated in both animal models and the cultured human mesothelial cell line. Nine intersection genes among baicalein targets and the human peritoneum RNA-seq dataset including four encapsulating peritoneal sclerosis samples and four controls were predicted by network analysis. Among them, MMP2, BAX, ADORA3, HIF1A, PIM1, CA12, and ALOX5 exhibited higher expression in the peritoneum with encapsulating peritoneal sclerosis compared with those in the control, which might be crucial targets of baicalein against peritoneal fibrosis. Furthermore, KEGG and GO enrichment analyses suggested that baicalein played an anti-peritoneal fibrosis role through the regulating cell proliferation, inflammatory response, and AGE-RAGE signaling pathway. Moreover, molecular docking analysis revealed a strong potential binding between baicalein and MMP2, which was consistent with the predictive results. Importantly, using a mouse model of peritoneal fibrosis by intraperitoneally injecting 4.25% glucose dialysate, we found that baicalein treatment significantly attenuated peritoneal fibrosis, as evident by decreased collagen deposition, protein expression of *α*-SMA and fibronectin, and peritoneal thickness, at least, by reducing the expression of MMP2, suggesting that baicalein may have therapeutic potential in suppressing peritoneal dialysis-related fibrosis.

## Introduction

Peritoneal dialysis (PD) is an important alternative therapy for patients with end-stage renal disease (See et al., 2018). Effective PD depends on structural and functional integrities of the peritoneal membrane, but long-term PD may cause progressive peritoneal membrane fibrosis or even encapsulating peritoneal sclerosis (EPS), ultimately resulting in ultrafiltration failure and increased mortality (Morelle et al., 2015). EPS is characterized by intraperitoneal inflammation and marked sclerotic thickening of the peritoneum, as well as systemic malnutrition accompanied by intestinal adhesion and obstruction (Johnson et al., 2010). Although the exact mechanism remains unclear, accumulating evidence has suggested that peritoneal fibrosis occurs in response to a cascade of injuries, including continuous exposure to bioincompatible dialysate (Davies et al., 2001), uremic toxins (Williams et al., 2002), and peritonitis (Davies et al., 1996). Upon these stimulations, inflammation (Balzer et al., 2019), neoangiogenesis (Kariya et al., 2018), and mesothelial-to-mesenchymal transition (MMT) (Chen et al., 2014) are thought to be crucial pathological changes and ultimately result in discontinuation of PD treatment (Zhou et al., 2016). Multiple therapeutic regimes for PD-associated peritoneal fibrosis have been trialed, but most of them have been animal studies and further clinical validation is lacking (Nakayama et al., 2017; Herzog et al., 2021). Thus, we identified differentially expressed genes (DEGs) in PD patients with EPS and performed target-based screening by network pharmacology to explore the anti-fibrotic compound targeting EPS.

Baicalein (5,6,7-trihydroxyflavone; BAI) is a flavonoid derived from the root of *Scutellaria baicalensis* and has a long history of use (Li et al., 2014). Baicalein has anti-inflammatory, antioxidant, anti-cancer, and anti-fibrotic effects (Bie et al., 2017; Liang et al., 2017). Among the five extracts of *S. baicalensis*, baicalein exhibits the highest anti-fibrotic efficacy (Zhou et al., 2022). Additionally, baicalein ameliorates fibrosis in multiple organs, including the heart, lung, liver, and kidney, by regulating inflammatory signaling pathways (Li et al., 2017; Lin et al., 2018). Baicalein is also safe and well tolerated in animal studies. Notably, baicalein transdermal administration significantly attenuates skin hypertrophic scar formation and collagen deposition in a mechanical load-induced mouse model (Zhang et al., 2016), suggesting that baicalein acts on the skin surface. The peritoneum has the surface area and resembles the body surface (Lemoine et al., 2016). We, thus, hypothesized that baicalein may exert a therapeutic effect on peritoneal fibrosis via intraperitoneal injection.

Given multidirectional pharmacology and system biology, network pharmacology is an emerging subject of systematic drug research. The associations among a drug, target, and disease are comprehensively analyzed by data mining at the molecular level, and potential targets of a drug are predicted (Li et al., 2022a). To date, network pharmacology has been widely applied to explore the mechanism of compounds in traditional Chinese medicine (TCM).

**FIGURE 1:**
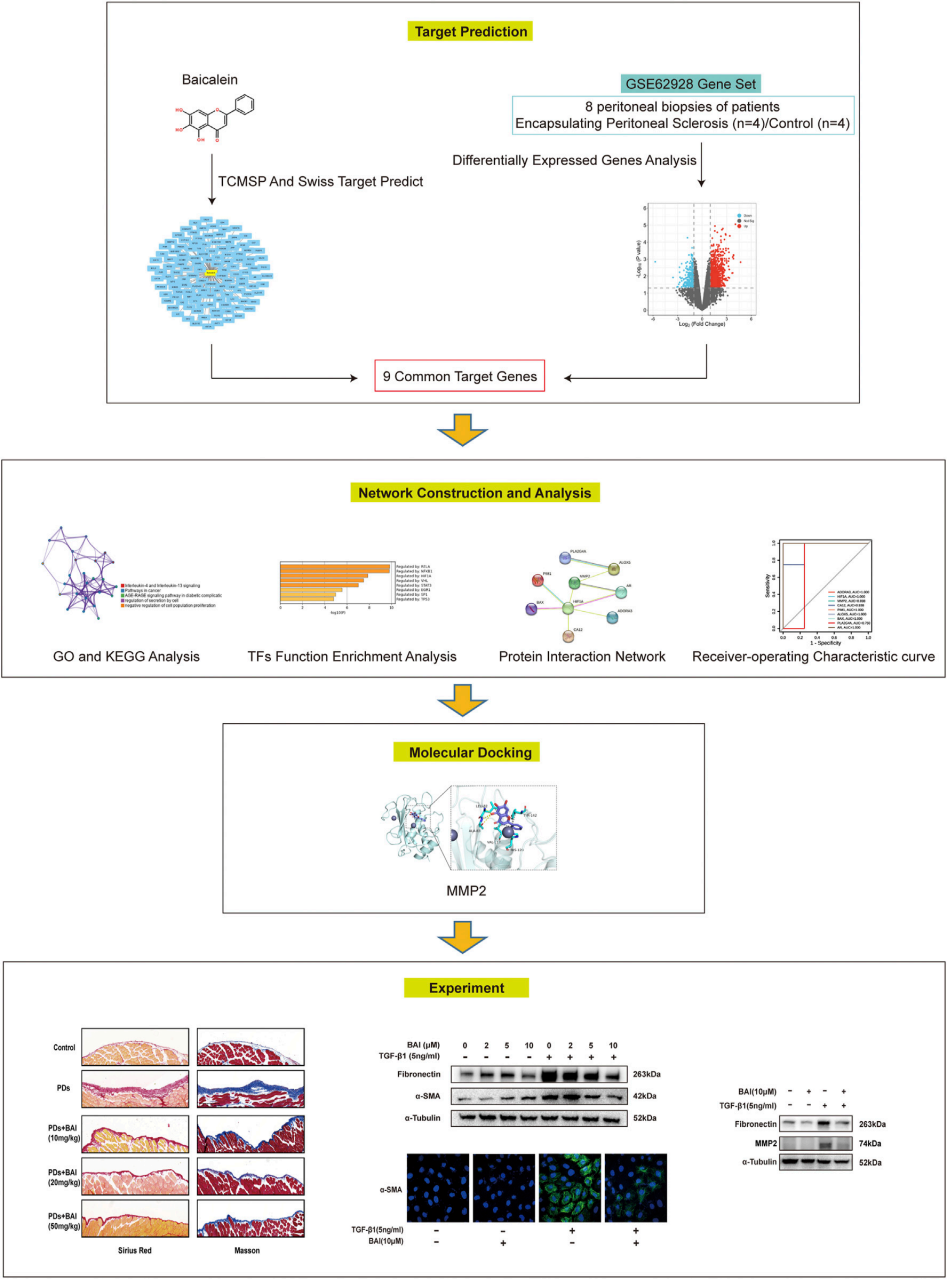
Flow chart of this study.

In this study, network pharmacology analysis was performed to investigate the role and mechanisms of baicalein in PD-associated peritoneal fibrosis. The prediction results were validated *in vitro* by the TGF-β1 treatment of the cultured human mesothelial cell line (MeT-5A cells) and *in vivo* using a mouse model of peritoneal fibrosis. The flowchart of this study is shown in Figure 1. This is the first report of the anti-fibrotic effect of baicalein in PD-associated peritoneal fibrosis via formulation with peritoneal dialysate as an intraperitoneal injection drug, which provides a new strategy for the development of novel dialysates to slow down peritoneal fibrosis.

## Methods and materials

### Data acquisition and DEG analysis

The human peritoneum RNA-seq dataset with four EPS samples from peritoneal biopsies of PD patients with EPS and four control (CTL) samples from uremia patients who underwent abdominal surgery for non-peritoneal conditions (*n* = 2) or first implantation of a peritoneal dialysis catheter (n = 2), published or updated in 2014–2018, were downloaded from the Gene Expression Omnibus database (GSE62928) using the GEO query package in R software, version 4.04 (Davis and Meltzer, 2007). DEGs between EPS and CTL were obtained using the limma package in R software (Ritchie et al., 2015). DEGs were defined as | log (fold change) |≥ 1 and *p* < 0.05. A volcano map and clustered heat map were generated using ggplot2 plotting and pheatmap library in R software, respectively (Gu et al., 2016; Jackson et al., 2018)^.^

### Baicalein target gene acquisition and common target gene analysis

The molecular structure of baicalein was retrieved from PubChem (https://pubchem.ncbi.nlm.nih.gov/). The traditional Chinese medicine systems pharmacology (TCMSP) database and the SwissTargetPrediction and analysis platform (Ru et al., 2014) were used to determine baicalein target genes. CTGs of baicalein among EPS-related DEGs (EPS-DEGs) were identified using the Venn diagram R package. To better understand the interactions of DEGs, a Search Tool for the Retrieval of Interacting Genes/Proteins (STRING) was performed to construct a protein–protein interaction (PPI) network for CTGs (Kathuria et al., 2020). PPI networks were visualized by Cytoscape v3.5.1. The expression condition of CTGs in EPS was visualized by a clustered heat map using complex pheatmap library in R software. The receiver operating characteristic (ROC) analysis was performed in R using the pROC software package to calculate the cutoff values of CTGs, and AUC was used to quantify the diagnostic accuracy.

### Metascape common target gene list analysis

For the CTG list, pathway and process enrichment analysis was carried out with the following ontology sources: Kyoto Encyclopedia of Genes and Genomes (KEGG) pathway, Gene Ontology (GO) Biological Processes, Reactome Gene Sets, Canonical Pathways, Collection of experimentally verified mammalian protein complexes, and Wiki Pathways using Metascape (https://metascape.org) (Chen et al., 2021). Transcriptional Regulatory Relationships Unraveled by Sentence-based Text-mining was used to infer regulatory interactions between human transcription factors and CTGs (Han et al., 2018). All genes in the genome were used as the enrichment background. Terms with *p*-value < 0.01, a minimum count of 3, and enrichment factor > 1.5 (enrichment factor is the ratio between the observed count and the count expected by chance) were collected and grouped into clusters by their membership similarities. To further capture the relationships between the terms, a subset of enriched terms was selected and rendered as a network plot, where terms with similarity >0.3 were connected by edges. The network was visualized using Cytoscape v3.5.1.

### Molecular docking of baicalein components

The three-dimensional (3D) structure of baicalein was obtained from the PubChem database. The structure was minimized with the MMFF94 force field (Anderson et al., 2017). All crystal structure coordinates were downloaded from the protein data bank (http://www.rcsb.org/) or AlphaFold database (https://alphafold.ebi.ac.uk) (Trott et al., 2010). All water, hydrogen, and ligand molecules were removed from the crystal structure using PyMol 2.5 software prior to docking. The docking site was defined to cover the entire active site. Both the ligand and receptor were converted to the PDBQT format using ADFRsuite 1.0. Docking calculations were performed using AutoDock Vina 1.1.2 (http://vina.scripps.edu/) with exhaustiveness set at 32 (Sahoo et al., 2018). We selected the conformation with the highest total score, visualized, and analyzed with PyMOL 2.5 (DeLano Scientific LLC, San Carlos, CA, United States).

### Animal model

Male C57/BL6 mice (aged 8–12 weeks) weighing 25–30 g was purchased from Jiangsu Gem Pharmatech Biotechnology Company (Jiangsu, China). All animal experiments were performed under the approval of the Institutional Animal Care and Use Committee of Sun Yat-Sen University. Mice were randomly divided into five groups (*n* = 5). 1) Control treated with vehicle; 2) control treated with BAI (10, 20, and 50 mg/kg); 3) peritoneal fibrosis (PF) treated with vehicle; 4) PF treated with 10 mg/kg BAI; 5) PF treated with 20 mg/kg BAI; and 6) PF treated with 50 mg/kg BAI. Baicalein (MCE, HY-N0196, United States) was dissolved in dimethyl sulfoxide (Sigma, D2650, United States) and then diluted into different concentrations with the 4.25% dextrose PD solution (Baxter, United States). A mice model of peritoneal fibrosis was induced by intraperitoneal injection of the 4.25% dextrose PD solution for 6 weeks (Si et al., 2019) with or without baicalein mixed. In the control group, mice were administered with saline.

### Cell culture

MeT-5A cells were purchased from the American Type Culture Collection (ATCC CRL-9444, United States) and were cultured at 37 C with 5% CO_2_ in Medium 199 containing 1.5 g/L sodium bicarbonate, 10% fetal bovine serum, 3.3 nM epidermal growth factor, 400 nM hydrocortisone, 870 nM zinc-free bovine insulin, and 20 mM HEPES. At 70% confluence, the culture medium was replaced with serum-free medium for 12 h. Cells were then treated with TGF-β1 (R&D, 240-B-002, United States) 5 ng/mL with or without baicalein (MCE, HY-N0196, United States) 1, 2, 5, 10, and 20 μM for 24 h.

### Western blotting

Cells were lysed in the RIPA buffer (Merck Millipore, 20-188, Germany) with complete protease inhibitors (Roche, 5892791001, Swiss) and PhosSTOP phosphatase inhibitor (Roche, 490683700, Swiss). Lysates were centrifugated at 12, 000 rpm for 15 min at 4 C. Supernatants were collected, and the protein concentration was measured using a BCA Protein Assay Kit (Beyotime, P0012, China). Proteins were separated in SurePAGE, Bis-Tris, 10%15 wells (GeneScript, M00666, United States) and transferred onto PVDF membranes (Merck Millipore, IPVH00010, Germany). Then, 5% bovine serum albumin was used to block the membranes for 1 h, followed by incubation with primary antibodies overnight at 4 C, including the anti-fibronectin antibody (1:100, Boster, BM1772, China), anti-α-SMA antibody (1:100, Sigma, 5228, United States), anti-MMP2 antibody (1:100, abcam, ab92536, United Kingdom), anti-TIMP2 antibody (1:100, Santa cruz, sc21735, United States), anti-GAPDH antibody (1:500, CST, 2118s, United States), and anti-αTubulin antibody (1:100, CST, 12351s, United States). Corresponding secondary antibodies were applied at 37 C for 1 h.

### Real-time quantitative polymerase chain reaction

Total RNA was extracted from cells using an RNeasy Mini Kit (Qiagen, 74,104, Germany), following the manufacturers’ protocol. cDNA was synthesized from 1 μg total RNA using HiScriptII QRT SuperMix for qPCR (Vazyme, R223-01, China). qPCR was performed using ChamQ Universal SYBR qPCR Master Mix (Vazyme, Q711-02, China) with QuantStudio™ Design & Analysis Software v1.4.3. Primers sequences were showed in Supplementary Table S1.

### Histology and immunohistochemistry

The peritoneal membrane was fixed in 4% paraformaldehyde, soaked in 30% sucrose overnight, embedded in paraffin, and cut into 6 μm-thick sections. Sirius Red and Masson staining was performed to evaluate peritoneal fibrosis. The thickness of the submesothelial compact zone was determined under a microscope (Zeiss LM; Zeiss, Germany) using Image-Pro Plus 6.0 software. The peritoneal thickness was calculated by the average of the collagen area/peritoneum length.

For immunohistochemistry, sections were deparaffinized and the endogenous peroxidase activity was quenched in 3% H_2_O_2_ for 15 min. Non-specific antigens were blocked in serum for 60 min at 37 C. The sections were then incubated with indicated primary antibodies, including the anti-α-SMA antibody (1:100, Sigma, 5228, United States) and anti-Collagen Ⅰ antibody (1:100, Boster, PB0325, China), overnight at 4 C. Sections were then treated with HRP-conjugated corresponding secondary antibodies. Florescence microscopy was performed under the Zeiss LM microscope. The immunohistochemically stained area was quantitatively measured using Image-Pro Plus 6.0 software.

### Immunofluorescence

MeT-5A cells were fixed in 4% paraformaldehyde at 37 C for 15 min, permeabilized in 0.25% Triton X-100 (Sigma, V900502, United States) for 10 min, washed for 5 min with PBS three times, incubated with 5% bovine serum albumin for 30 min, and then, incubated with mouse anti-α-SMA (1:100, Sigma, 5228, United States) antibodies overnight at 4 C in a humidified atmosphere. Then, cells were washed three times with PBS for 5 min, incubated with FITC-conjugated secondary antibodies at 37 C for 1 h, and then, counterstained with DAPI for 5 min. Fluorescence microscopy was performed to analyze the biomarker expression. At least three biological repeats were performed.

### Cell viability assay

Cell viability was examined using an MTS assay kit (Promega, United States). In brief, RPMI (100 μL) was mixed with the 20 μL/well MTS solution, cells were incubated for 2 h, and then, absorbance at 490 nm was recorded using a 96-well plate reader (Bio-Tek, Winooski, VT).

### Statistical analysis

Data were expressed as the mean ± SEM. Student’s t-test was used to determine significant differences between two groups, and one-way analysis of variance (ANOVA) followed by Tukey’s posttest for multiple comparisons was used for groups of three or more. A value of *p* < 0.05 was considered statistically significant.

## Results

### Data acquisition and DEG identification

**FIGURE 2:**
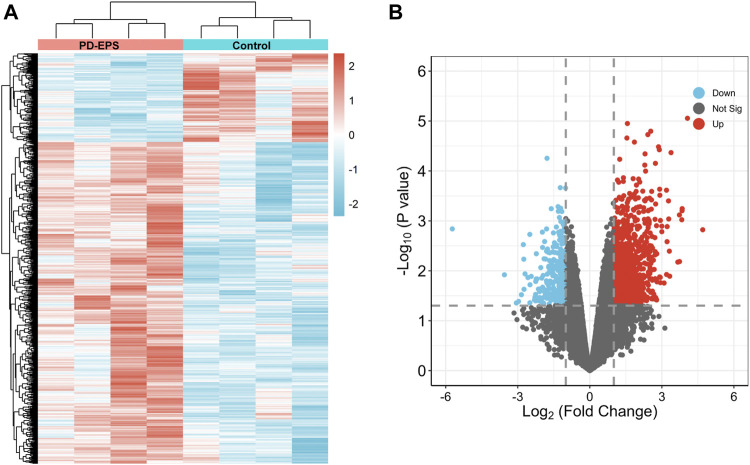
DEG identification. A heat map **(A)** and volcano plot **(B)** showing 1,057 DEGs. Red indicates upregulated genes, and blue indicates downregulated genes. *p* < 0.05, |log2(FC)|>1.

To identify DEGs in patients of PD-related EPS, we analyzed the GSE62928 gene set from peritoneal biopsies of four patients with EPS (EPS group) obtained after 74 ± 33.52 months of PD and four patients with uremia untreated with peritoneal dialysis (CTL group). The baseline clinical characteristics are detailed in DOI: 10.1371/journal.pone.0056389.A box plot showed the median of each sample on a horizontal line, indicating good normalization between the samples (Supplementary Figure S1). Next, we identified DEGs with the criteria of | log (fold change) | ≥1 and *p* < 0.05 (Supplementary Excel File 1). A heatmap of the 1,057 DEGs by cluster is presented in Figures 2A, and a volcano map showed that the expression of upregulated genes and downregulated genes in the EPS group as compared with the CTL group (Figure 2B).

### Baicalein targets and intersects with EPS-associated DEGs

**FIGURE 3:**
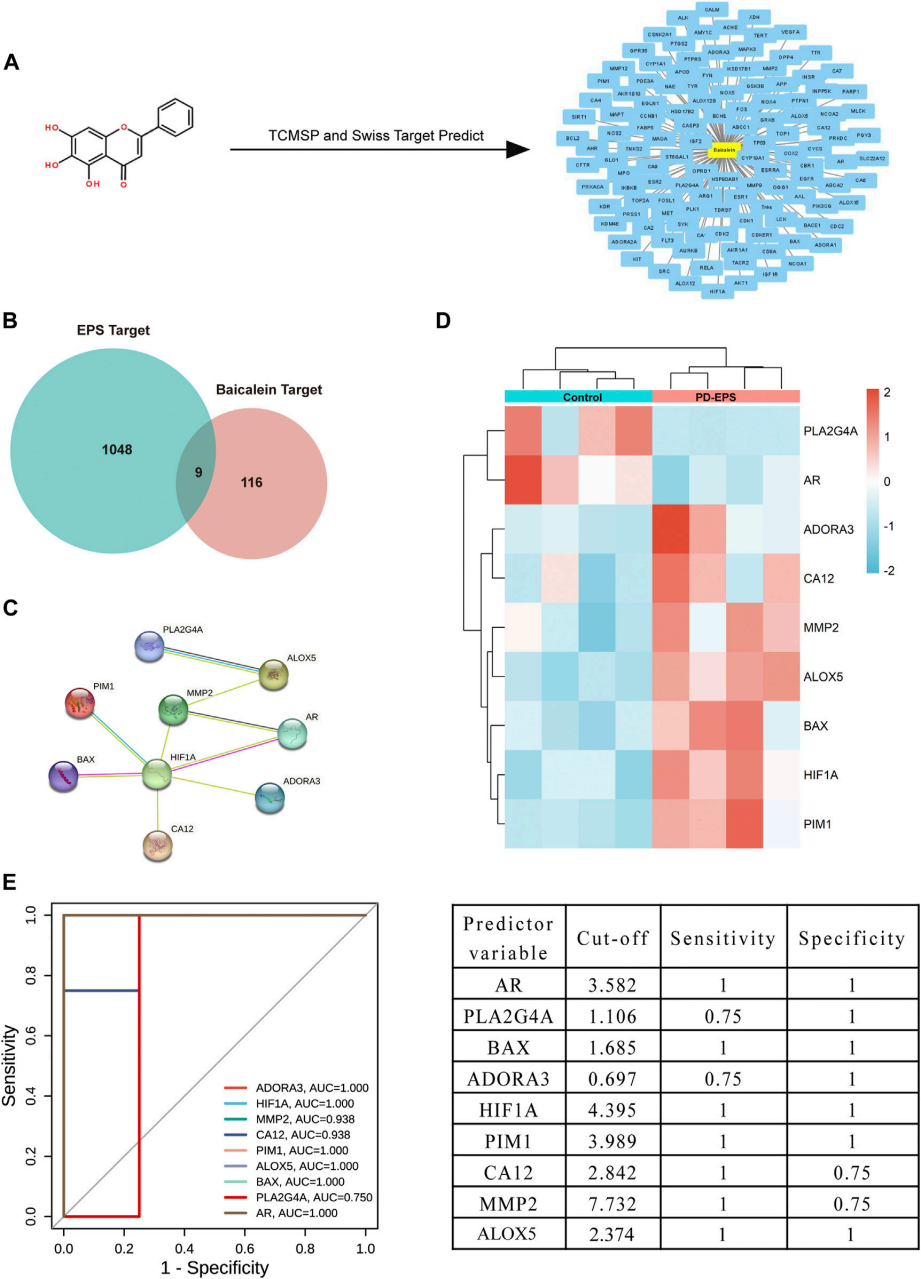
Common targets of EPS-DEGs and baicalein. **(A)** Baicalein target network. The node in yellow is baicalein, and nodes in blue are candidate targets of baicalein; **(B)** Venn diagram of nine CTGs among EPS-DEGs and baicalein; **(C)** CTGs in the PPI network; **(D)** heat map showing CTG expression in the PD patients with EPS compared with that in the CTL; and **(E)** ROC curve of CTGs as predictor diagnostic biomarkers of PD-EPS.

Inflammation or fibrosis improvement by baicalein has been reported previously (Wang et al., 2015a; Bie et al., 2017; Liang et al., 2017). Therefore, we compared common target genes (CTGs) of baicalein and EPS-DEGs. A total of 125 target genes of baicalein were obtained by TCMSP and SwissTargetPrediction (Figure 3A) and converted into symbols of corresponding genes using UniProt (Supplementary Excel File 2). Additionally, nine intersecting target genes were identified among baicalein targets and EPS-DEGs (Figure 3B) that were further explored by the PPI analysis. The PPI network of these nine common genes yielded significant gene interactions using the STRING database (Figure 3C). Moreover, BAX, ADORA3, HIF1A, PIM1, CA12, MMP2, and ALOX5 exhibited low expression in the CTL group and high expression in the EPS group (Figure 3D). Combined with the results of PPI, these genes may be major therapeutic targets of baicalein. We validated these results through the receiver operating characteristic (ROC) curve analysis to evaluate the diagnostic performance of the nine CTGs for PD-related EPS. As shown in Figure 3E, all area under curve (AUC) values of CTGs except for PLA2G4A and ADORA3 were more than 0.9 and exhibited high sensitivity and specificity. This suggests that CTGs had a superior ability as predictive diagnostic biomarkers for PD-related EPS.

### Functional enrichment analysis of CTGs

**FIGURE 4:**
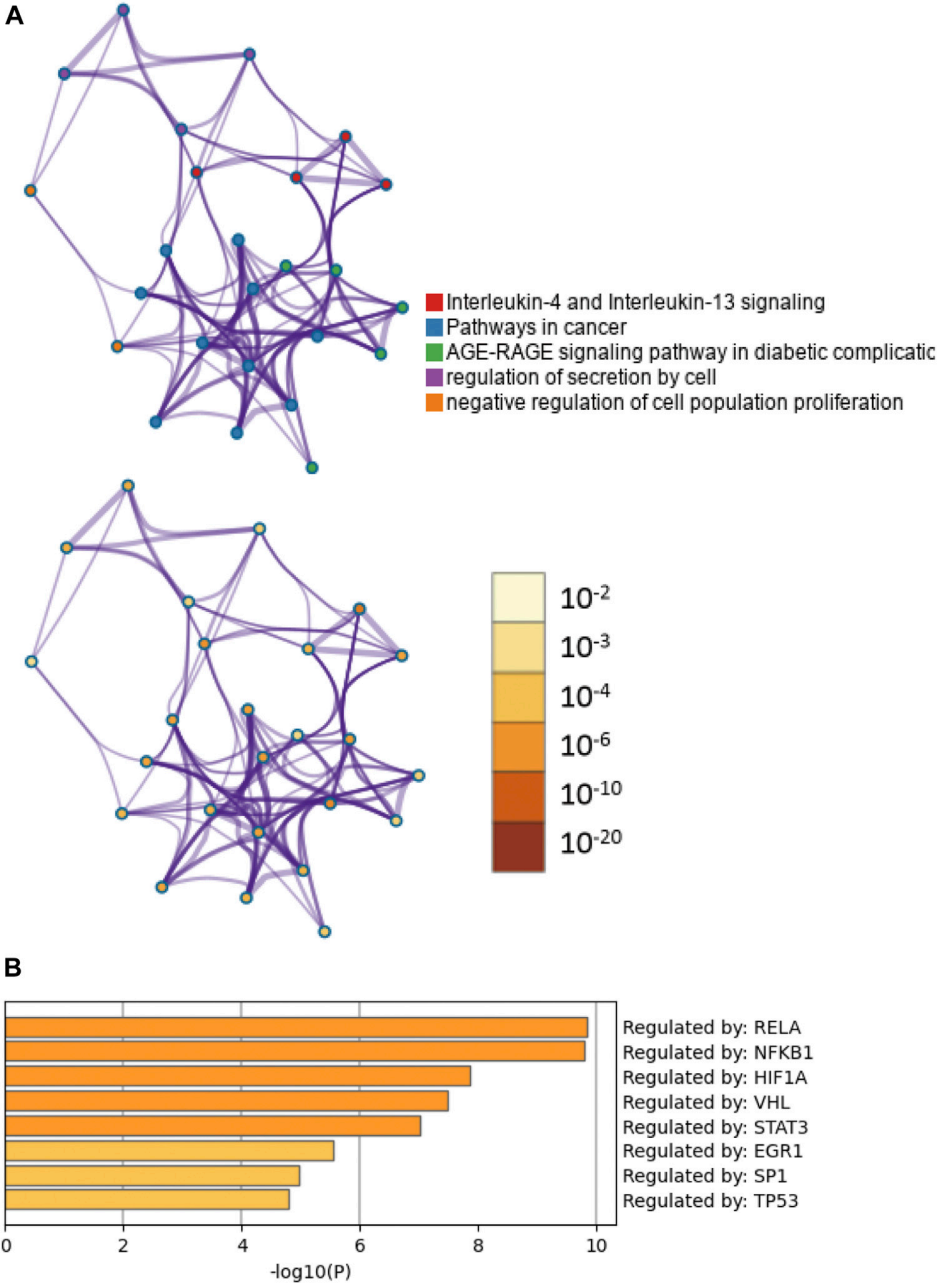
Functional enrichment and transcription factor analyses of common target genes. **(A)** The network of enriched pathways and processes in the figure above are colored by cluster ID; nodes that have the same cluster ID are typically close to each other. The enrichment network at the bottom of the figure is colored by *p*-value; terms containing more genes tend to have a more significant *p*-value. **(B)** TF functional enrichment analysis of common targets.

To clarify the potential biological functions of nine CTGs, further analysis was performed using Metascape. The pathway and process enrichment analysis revealed that these CTGs were closely associated with cell proliferation and inflammation including interleukin-4 and interleukin-13 signaling, AGE-RAGE signaling, cancer pathways, regulation of epithelial cell proliferation, regulation of cell secretion, and the inflammatory response (Figure 4A; Supplementary Table S2; Supplementary Excel File 3). These pathways and processes exhibited significantly overlap or intersected with each other (Figure 4A). Moreover, these CTGs were transcriptionally regulated by RELA, NFKB1, and HIF-1 (Figure 4B; Supplementary Table S3). These results implied that baicalein might improve peritoneal fibrosis by regulating CTG-mediated pathways and processes.

### Molecular docking analysis

**FIGURE 5:**
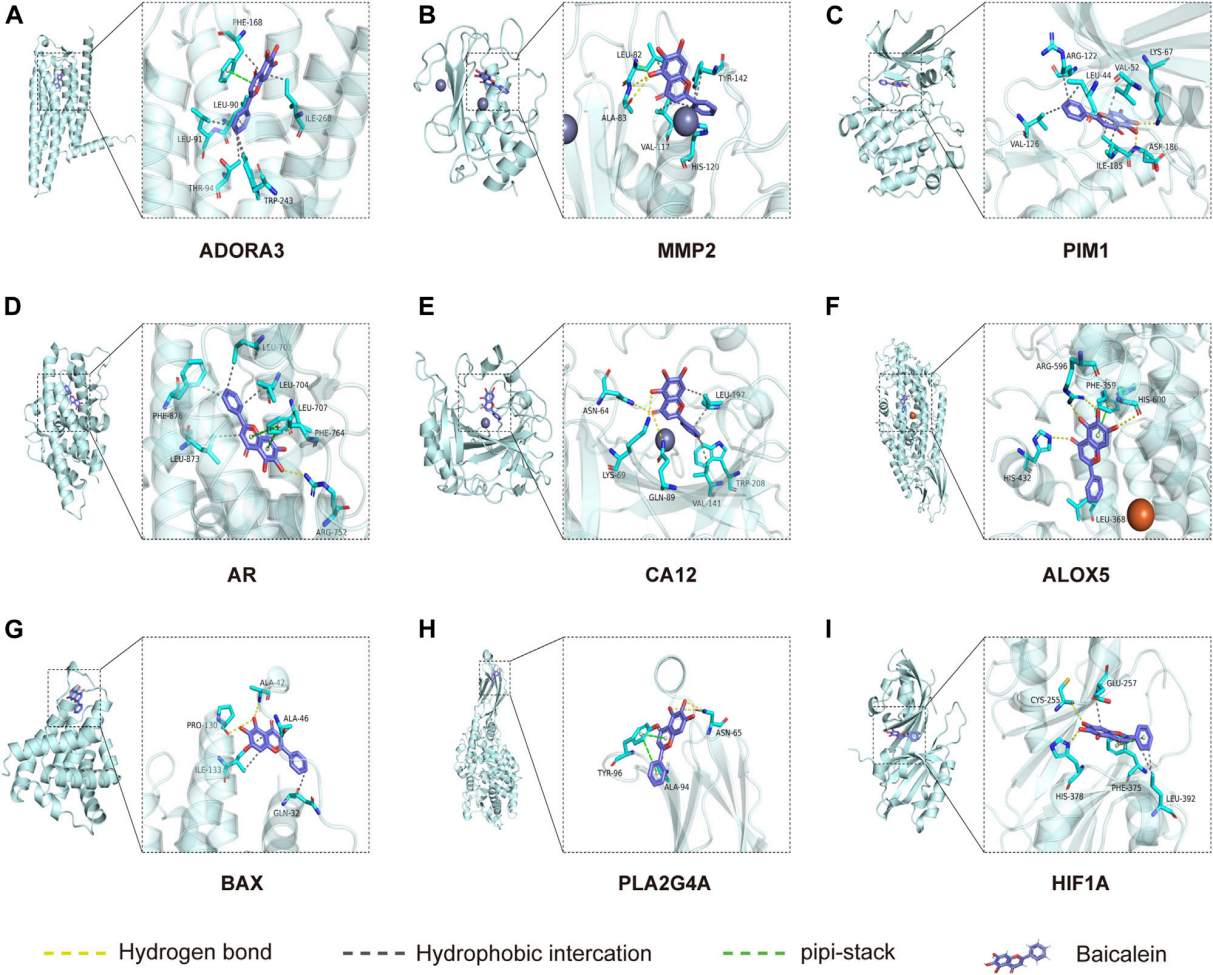
Molecular docking between baicalein and common targets. **(A)** ADORA3–baicalein; **(B)** MMP2–baicalein; **(C)** PIM1–baicalein; **(D)** AR–baicalein; **(E)** CA12-Baicalein; **(F)** ALOC5–baicalein; **(G)** BAX–baicalein; **(H)** PLA2G4A–baicalein; and **(I)** HIF1A–baicalein.

Molecular docking is used to explore the mode and strength of biomolecular interactions at the molecular level (Li et al., 2022a). Here, we interpreted the binding strength and mechanism of baicalein and nine CTGs through molecular docking using the AutoDock Vina program. A negative binding affinity indicates a binding possibility, and binding energy of ≤−6 kcal/mol is generally considered more likely for binding. All crystal structure coordinates are shown in Supplementary Table S4. We obtained the binding energy of each target with baicalein (Table 1) and observed a strong binding potential between baicalein and target genes. The 3D complex structure of baicalein-common targets was reconstructed and optimized as shown in Figure 5. The 3D interaction map of the best ranking poses showed that baicalein formed some favorable interactions and docked comfortably within the catalytic region of CTGs, suggesting that CTGs have potential to serve as targets of baicalein. Among them, ALOXA3, PIM1, and MMP2 showed strong binding potential with baicalein.

**TABLE 1:** Docking scores of baicalein and common targets.

Target name	Ligand name	Docking score
ADORA3	Baicalein	−9
MMP2	Baicalein	−8.6
PIM1	Baicalein	−8.5
AR	Baicalein	−8.2
CA12	Baicalein	−8.1
ALOX5	Baicalein	−7.5
BAX	Baicalein	−7.5
PLA2G4A	Baicalein	−6.4
HIF1A	Baicalein	−6.3

### Baicalein attenuated PD-associated peritoneal fibrosis *in vivo*

**FIGURE 6:**
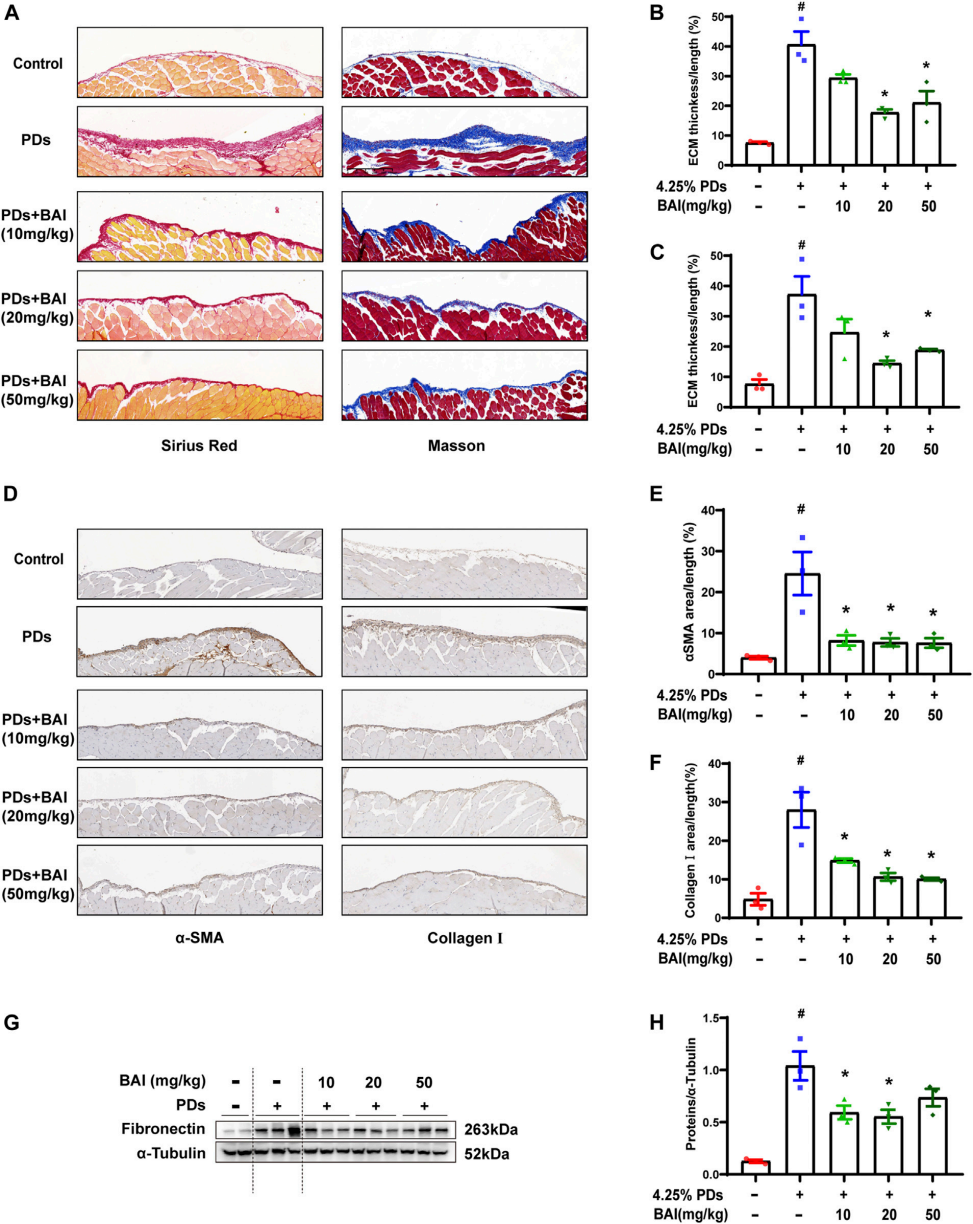
Baicalein attenuated PD-associated peritoneal fibrosis in mice. **(A)** Sirius Red and Masson staining of parietal peritoneum from mice treated with PDs plus baicalein at various concentrations; **(B)** quantitative analyses of the peritoneal thickness by Sirius Red; **(C)** quantitative analyses of the peritoneal thickness by Masson staining; **(D)** immunohistochemical detection of *α*-SMA and collagen Ⅰ in parietal peritoneum biopsies from mice treated with PDs plus baicalein at various concentrations; **(E)** quantitative analyses of *α*-SMA; **(F)** quantitative analyses of collagen Ⅰ; **(G)** immunoblot analyses of fibronectin, in the visceral peritoneum compared with the sham control; and **(H)** densitometric analyses of fibronectin in immunoblots. *α*-Tubulin was used as a loading control. Data in b, c, d, f, and h are means ± SEM (*n* = 3 per group). Symbols indicate individual animals, and lines indicate median values of the group. The 95% CI is indicated by the vertical error bar. ^#^ *p* < 0.05 vs. sham groups; *p < 0.05 vs. PDs groups.

Based on the prediction of network pharmacology analysis, we explored whether the baicalein treatment attenuated PD-associated peritoneal fibrosis in mice. To this end, mice were treated with daily intraperitoneal injections of the high-glucose PD solution (PDs) with or without 10, 20, and 50 mg/kg baicalein, while the control group was exposed to saline. Pathological staining revealed that the parietal peritoneum in mice exposed to PDs had a noticeably thickened submesothelial zone with more collagen deposition at 6 weeks after modeling, which was alleviated by baicalein (Figures 6A–C). Immunohistochemistry results showed that baicalein treatment significantly reduced PDs-induced expression of *α*-SMA and Collagen I compared with the PDs group (Figures 6D–F). Furthermore, immunoblot analysis demonstrated marked attenuation of PDs-induced expression of fibronectin by baicalein (Figures 6G, H). Notably, no dose-dependent effect of baicalein was observed among the three dosages (10, 20, and 50 mg/kg). Collectively, consistent with the pharmacological prediction, baicalein conferred a protection against PD-associated peritoneal fibrosis in mice.

### Baicalein inhibited the production of the extracellular matrix *in vitro*

**FIGURE 7:**
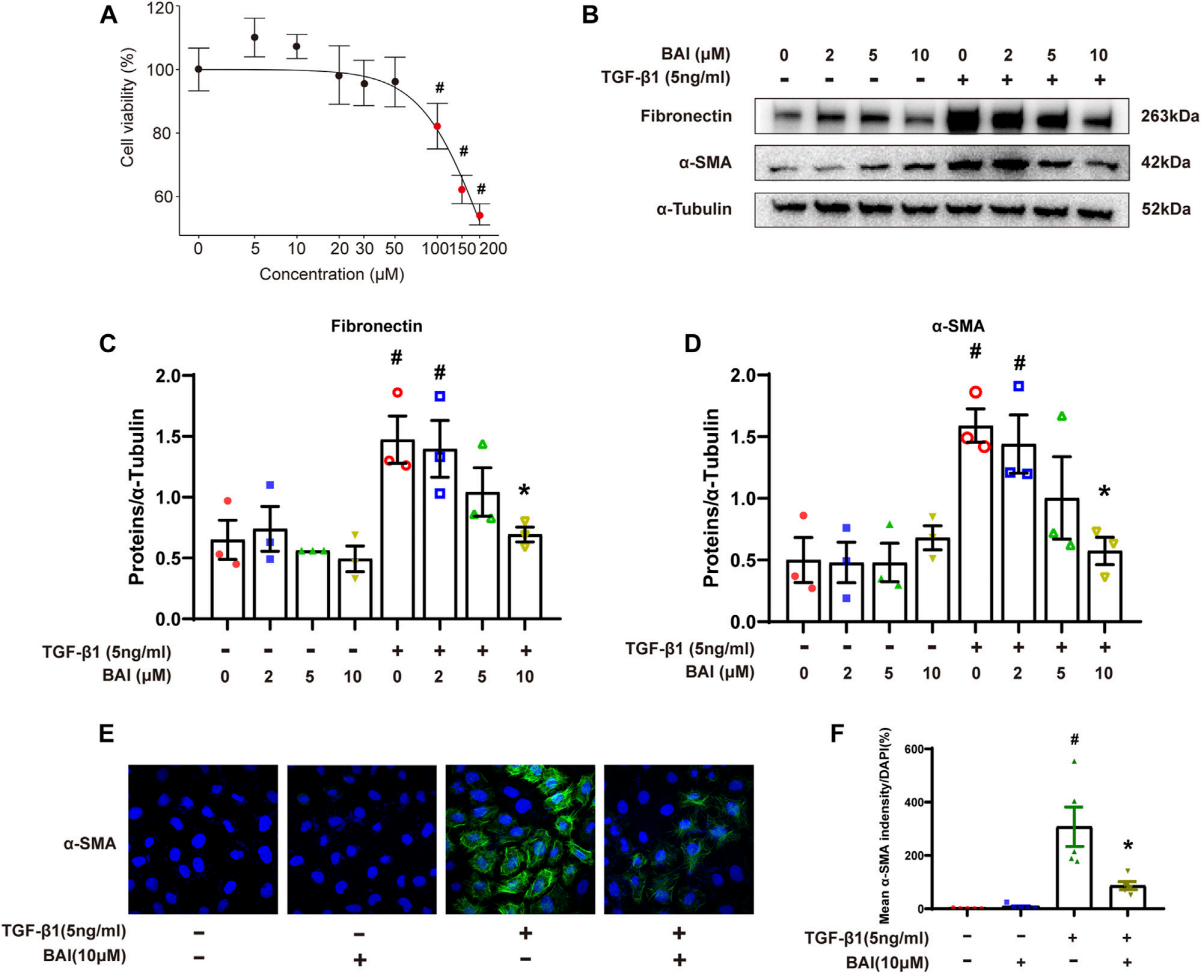
Baicalein inhibited the production of the extracellular matrix *in vitro*. **(A)** Cytotoxicity assays (MTS) to evaluate the viability of MeT-5A cells incubated with 0–200 μM baicalein. Mean ± SE (*n* = 3); **(B)** immunoblot analyses of fibronectin and *α*-SMA in MeT-5A cells with TGF-β1 and baicalein at different concentrations; **(C)** densitometric analyses of fibronectin in immunoblots; *α*-Tubulin was used as a loading control; **(D)** densitometric analyses of *α*-SMA in immunoblots; *α*-Tubulin was used as a loading control; **(E)** immunofluorescence staining of *α*-SMA (green) and DAPI staining (blue) of MeT-5A cells after treatment with TGF-β1 (5 ng/mL) and DMSO or baicalein for 24 h; and **(F)** quantitative analysis of immunofluorescence staining of *α*-SMA. Data in d, f are means ± SEM (*n* = 3 per group). Symbols indicate individual samples, and lines indicate median values of the group. The 95% CI is indicated by the vertical error bar. ^#^ *p* < 0.05 vs. control groups and *p < 0.05 vs. TGF-β1 groups.

We next investigate the effects of baicalein on TGF-β1-induced production of the extracellular matrix (ECM) in human mesothelial cells (MeT-5A) *in vitro*. MTS assay revealed that baicalein treatment for 24 h significantly decreased cell viability at concentration above 100 μM (Figure 7A). After being treated with various concentration of baicalein, increased expression of fibronectin and *α*-SMA induced by TGF-β1 was significantly alleviated in a dose dependent manner (Figures 7B–D). Immunofluorescence staining of *α*-SMA showed similar results (Figures 7E, F). These results verified that baicalein directly protected mesothelial cells from TGF-β1-induced production of ECM.

### Baicalein downregulated the expression of MMP2 both *in vitro* and *in vivo*

**FIGURE 8:**
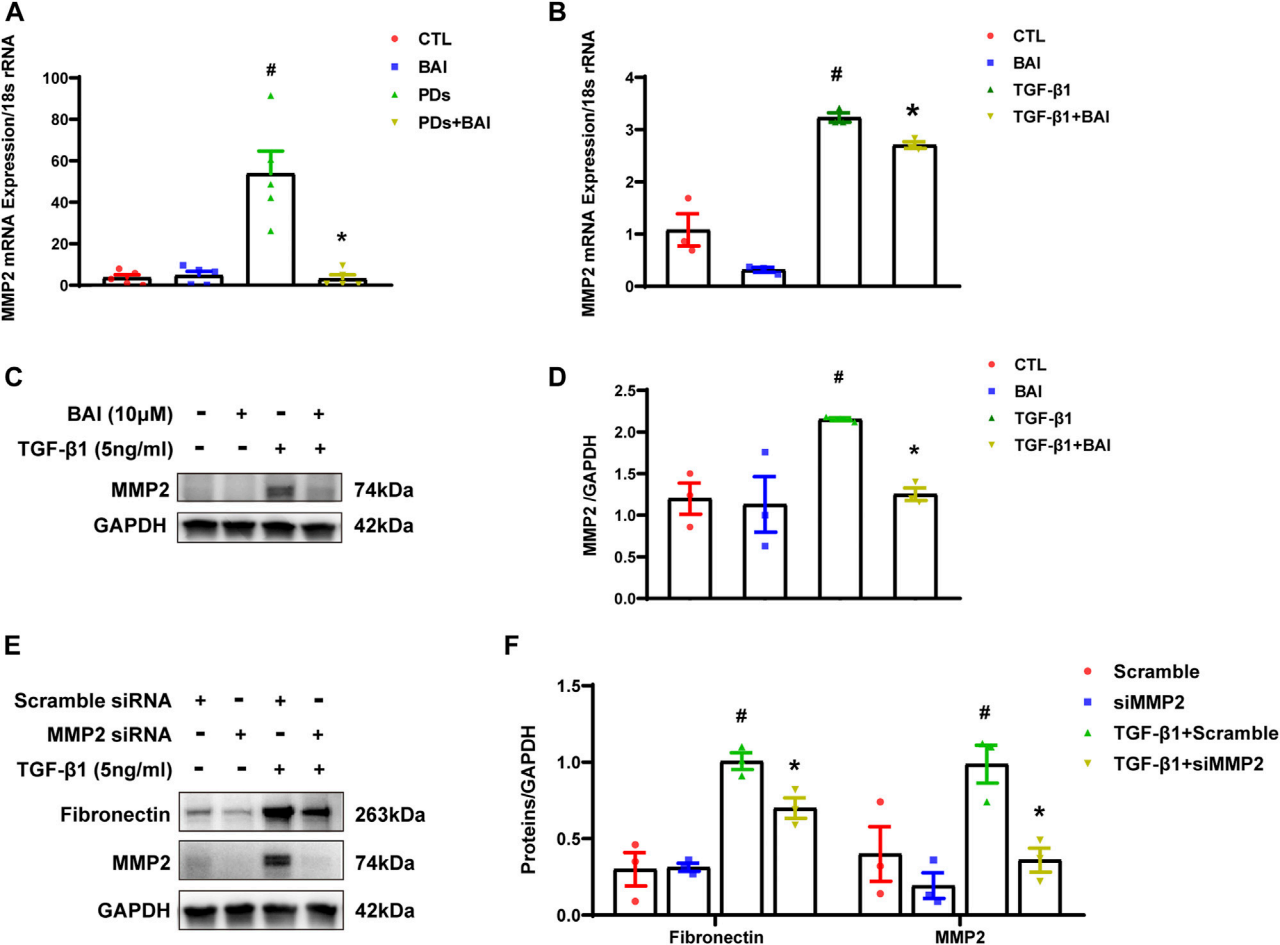
Baicalein downregulated the expression of MMP2 both *in vitro* and *in vivo*. **(A)** RT-qPCR analysis of MMP2 mRNA expression in visceral peritoneum biopsies from mice treated with PDs plus baicalein. 18S-rRNA was used as a loading control; **(B)** RT-qPCR analysis of MMP2 mRNA expression in MeT-5A cells. 18S-rRNA was used as a loading control; **(C)** immunoblot analyses of MMP2 in MeT-5A cells; **(D)** densitometric analyses of MMP2 in immunoblots. GAPDH were used as a loading control; **(E)** immunoblot analyses of fibronectin, MMP2 in MeT-5A cells with various treatments; and **(F)** densitometric analyses of fibronectin, MMP2 in immunoblots. GAPDH were used as a loading control. Data in a, b, d, and f are means ± SEM (n = 3 per group). Symbols indicate individual samples, and lines indicate median values of the group. 95% CI is indicated by the vertical error bar. ^#^ *p* < 0.05 vs. control groups; *p < 0.05 vs. TGF-β1 groups.

To determine whether the predicted CTGs contributed to amelioration of peritoneal fibrosis, we performed qPCR analyses of the nine CTGs in the molecular docking analysis. mRNA expression of MMP2 displayed significant changes under TGF-β1 stimulation, but only elevated MMP2 was reversed by baicalein (10 μM) (Supplementary Figure S2). The expression level of MMP2 plays a significant role in protein hydrolysis, tissue repair, and the development of fibrosis (Zhang et al., 2022). Hence, we evaluated mRNA expression of MMP2 *in vivo and vitro*. Mice in PDs group displayed a significant upregulation of the MMP2 mRNA level, which was suppressed by treatment with baicalein (Figure 8A). Exposure of MeT-5A cells to TGF-β1 for 24 h, the mRNA expression of MMP2 was significantly increased, and this alteration was partially reversed by baicalein at both the mRNA (Figure 8B) and protein levels (Figures 8C, D). To address the role of MMP2 in peritoneal fibrosis, we silenced MMP2 by transfecting MMP2 siRNA. Upon TGF-β1 treatment, fibronectin was decreased in MMP2-silenced cells as compared with cells transfected with the empty vector, suggesting that the MMP2 level may contribute to the reduction of ECM (Figures 8E, F). Taken together, baicalein may exert anti-fibrotic effects through the downregulation of MMP2.

## Discussion

Peritoneal fibrosis is a critical complication of PD. The pathological alterations may lead to ultrafiltration failure and eventually to the discontinuation of therapy. Chronic exposure to the bioincompatible dialysate has demonstrated to result in the fibrosis of the peritoneal membrane, but there is lack of effective treatment strategies. Although the new peritoneal dialysis solution, such as icodextrin, has been demonstrated to slow down the occurrence of peritoneal fibrosis (Goossen et al., 2020), a study revealed that icodextrin may lead to early local inflammation and marked angiogenesis in the peritoneum (Schaefer et al., 2018). The addition of Ala-Gln to PDs protected the peritoneum, probably via the modulating IL-17 expression (Ferrantelli et al., 2016). Additionally, H_2_ (Nakayama et al., 2017) and LiCl (Herzog et al., 2021) have been studied as dialysate additives to preserve peritoneum integrity, whose safety, feasibility, and cost effectiveness need to be further investigated.

Previous studies have shown that intravenous administration of baicalein significantly improves multi-organ fibrosis, suggesting its anti-fibrotic effect (Sun et al., 2010; Wang et al., 2015b). Moreover, baicalein administered intradermally attenuates hypertrophic scar formation, demonstrating direct absorption of baicalein by penetrating the skin barrier to exert an anti-fibrotic effect (Zhang et al., 2016). The peritoneum as a serosal membrane lining in the abdominal cavity consists of mesothelial cells and connective tissue, which suffers from repeatedly stimulation by high-glucose peritoneal dialysate and glucose metabolites. However, it is unclear whether baicalein has a therapeutic effect on PD-associated peritoneal fibrosis.

In this study, we attempted to explore the protective role of baicalein in both a mice model of peritoneal fibrosis and the cultured human mesothelial cells. We found that treatment with baicalein resulted in remarkably decreased extracellular matrix deposition and improved thickening of the peritoneum in mice, as compared with those treated with vehicle. Additionally, baicalein suppressed TGF-β1-induced ECM secretion of mesothelial cells. Our results indicated that baicalein has a great clinical potential and might be a therapeutic strategy for peritoneal fibrosis.

To investigate how baicalein protected the peritoneum from fibrosis, network pharmacological analysis was performed to identify nine common genes among baicalein targets and EPS-related DEGs. The PPI network showed that many important proteins of biological pathways were involved in baicalein-mediated therapeutic processes in PD-associated peritoneal fibrosis. HIF1A widely participates in the pathogenesis of fibrosis. A study by Yang et al. (2021) showed that activation of STAT3/HIF1A signaling mediated mesothelial to mesenchymal transition and ultimately alleviated peritoneal fibrosis. MMP2 expression increased after Chlorhexidine Gluconate-induced peritoneal fibrosis, which may be related to ECM degradation (Cui et al., 2022). BAX, PIM1, AR, and others are involved in signaling pathways such as apoptosis, cell migration, and metabolism which also involved in peritoneal fibrosis (Li et al., 2022b). The PPI indicated the complexity of peritoneal fibrosis and suggested that the therapeutic effects of baicalein involved multiple molecular mechanisms and network interactions of target proteins. GO and KEGG enrichment analysis further indicated that the most important mechanism of baicalein in protection against peritoneal fibrosis were probably related to cell secretion and inflammation.

Matrix metalloproteinases (MMPs) is a large family of zinc-requiring enzymes, and involved in several pathological processes such as migration, wound healing, inflammation, and fibrosis. Numerous studies have shown that MMPs exhibit low activity under normal conditions and increased activity during inflammation or repair (Santos et al., 2014). In CCl_4_-induced liver fibrosis, mRNA expression of MMP2 and MMP9 in the liver is elevated significantly (Zhang et al., 2022). Additionally, MMP7 mRNA expression is markedly upregulated in kidney tissues of chronic kidney disease patients (Zheng et al., 2022). At the early stage of renal fibrosis, MMP2 facilitates degradation of the glomerular baseline membrane and destroys the filtration barrier, which accelerates CKD progression (Cheng et al., 2022). However, in the late stage, the MMP2 activity is inhibited and ECM degradation decreases, promoting renal fibrosis (Ronco et al., 2007). A study showed that MMP2 alleviates kidney fibrosis in gene knockout mice (Tveitaras et al., 2015). Consistently, downregulating MMP2 by siRNA inhibited TGF-β1 stimulated the expression of *α*-SMA and fibronectin in cultured human mesothelial cells. Therefore, targeting MMP2 may attenuate the progression of peritoneal fibrosis during PD treatment.

In the present study, molecular docking analyses showed that MMP2 had great binding potential with baicalein. We validated the mRNA expression levels by RT-qPCR. Among nine DEGs, baicalein only reduced TGF-β1-induced MMP2 expression in cultured human mesothelial cells, indicating that MMP2 might be a crucial target of baicalein in alleviating peritoneal fibrosis. Indeed, in a mice model of high-glucose based dialysis solution-induced peritoneal fibrosis, we found that the mice on PD developed peritoneal fibrosis with increased MMP2 expression, which was reversed by baicalein addition to the PD solution.

There are some limitations in this study. The results were limited because of the small sample size obtained from a public database with only eight samples. Moreover, although nine CTGs were found and MMP2 was verified to be involved in mechanism of action, the underlying upstream or downstream relationship between MMP2 and baicalein needs to be explored. We also only studied the anti-fibrosis effects of baicalein in mesothelial cells and did not explore its role in regulating inflammatory. Therefore, further studies are required to elucidate the precise mechanisms by which baicalein inhibits the pathogenesis of peritoneal fibrosis.

## Conclusion

We demonstrated that baicalein had a marked anti-fibrotic effect on PD-associated peritoneal fibrosis by network pharmacology analysis and experimental validation. Its protective mechanism may be associated with a downregulation of MMP2, mediating collagen secretion and extracellular matrix deposition. This study presents the first use of baicalein as an additive of the PD solution, providing a new reference for the development of novel PDs.

## Data Availability

The datasets presented in this study can be found in online repositories. The names of the repository/repositories and accession number(s) can be found in the article/Supplementary Material.
