# Supplementary material for: Therapeutic mechanism of baicalein in peritoneal dialysis-associated peritoneal fibrosis based on network pharmacology and experimental validation
Source: Front Pharmacol. 2023 May 17;14:1153503. doi: 10.3389/fphar.2023.1153503 (PMC10229821; doi:10.3389/fphar.2023.1153503)
Supplement: Supplementary file 1 [file Table1.docx]

|  | Primer F (5’-3’) | Primer R (5’-3’) |
| --- | --- | --- |
| m-MMP2 | CCTGGACCCTGAAACCGTG | TCCCCATCATGGATTCGAGAA |
| m-18s rRNA | CCAACCTGGTTGATCCTGCCAGTA | CCTTGTTACGACTTCACCTTCCTCT |
| h-fibronectin | CGGTGGCTGTCAGTCAAAG | AAACCTCGGCTTCCTCCATAA |
| h-α-SMA | TGCCAACAACGTCATGTCG | CAGCGCGGTGATCTCTTTCT |
| h-ALOX5 | ACTGGCTGAATGACGACTGG | CAGGGGAACTCGATGTAGTCC |
| h-ADORA3 | GGCCAATGTTACCTACATCACC | CCAGGGCTAGAGAGACAATGAA |
| h-CA12 | AGTGAACGGTTCCAAGTGGAC | CCACACGACGGGTACTTCT |
| h-BAX | CCCGAGAGGTCTTTTTCCGAG | CCAGCCCATGATGGTTCTGAT |
| h-HIF1A | GAACGTCGAAAAGAAAAGTCTCG | CCTTATCAAGATGCGAACTCACA |
| h-AR | CCAGGGACCATGTTTTGCC | CGAAGACGACAAGATGGACAA |
| h-PLA2G4A | ATGGATGAAACTCTAGGGACAGC | CTGGGCATGAGCAAACTTCAA |
| h-PIM1 | GGCTCGGTCTACTCAGGCA | GGAAATCCGGTCCTTCTCCAC |
| h-MMP2 | CCCACTGCGGTTTTCTCGAAT | CAAAGGGGTATCCATCGCCAT |
| h-18s rRNA | GGCCGTTCTTAGTTGGTGGAGCG | CTGAACGCCACTTGTCCCTC |

**SUPPLEMENTARY TABLE 1 |Primers sequences.**

| **GO** | **Category** | **Description** | **Count** | **%** | **Log10(P)** | **Log10(q)** |
| --- | --- | --- | --- | --- | --- | --- |
| R-HSA-6785807 | Reactome Gene Sets | Interleukin-4 and Interleukin-13 signaling | 4 | 44.44 | -7.71 | -3.37 |
| hsa05200 | KEGG Pathway | Pathways in cancer | 5 | 55.56 | -6.71 | -2.66 |
| hsa04933 | KEGG Pathway | AGE-RAGE signaling pathway in diabetic complications | 3 | 33.33 | -5.53 | -1.93 |
| GO:1903530 | GO Biological Processes | regulation of secretion by cell | 4 | 44.44 | -4.84 | -1.58 |
| GO:0008285 | GO Biological Processes | negative regulation of cell population proliferation | 4 | 44.44 | -4.28 | -1.21 |

**SUPPLEMENTARY TABLE 2 |** **Top 5 clusters with their representative enriched terms (one per cluster).** "Count" is the number of genes in the user-provided lists with membership in the given ontology term. "%" is the percentage of all the user-provided genes that are found in the given ontology term (only input genes with at least one ontology term annotation are included in the calculation). "Log10(P)" is the p-value in log base 10. "Log10(q)" is the multi-test adjusted *p*-value in log base 10.

| **GO** | **Description** | **Count** | **%** | **Log10(P)** | **Log10(q)** |
| --- | --- | --- | --- | --- | --- |
| TRR01158 | Regulated by: RELA | 6 | 67 | -9.90 | -5.60 |
| TRR00875 | Regulated by: NFKB1 | 6 | 67 | -9.80 | -5.60 |
| TRR00484 | Regulated by: HIF1A | 4 | 44 | -7.90 | -4.40 |
| TRR01521 | Regulated by: VHL | 3 | 33 | -7.50 | -4.20 |
| TRR01277 | Regulated by: STAT3 | 4 | 44 | -7.00 | -3.80 |
| TRR00253 | Regulated by: EGR1 | 3 | 33 | -5.50 | -2.90 |
| TRR01256 | Regulated by: SP1 | 4 | 44 | -5.00 | -2.50 |
| TRR01419 | Regulated by: TP53 | 3 | 33 | -4.80 | -2.40 |

**SUPPLEMENTARY TABLE 3 |Summary of enrichment analysis in TRRUST.**

| **Target** | **PDB/AF ID** |
| --- | --- |
| HIF1A | 4h6j |
| MMP2 | 3ayu |
| BAX | 4s0o |
| PLA2G4A | 1CJY |
| PIM1 | 6YKD |
| CA12 | 7PUV |
| AR | 5V8Q |
| ALOX5 | 6N2W |
| ADORA3 | AF-P0DMS8 |

**SUPPLEMENTARY TABLE 4 | PDB/Aphafold ID of common target genes.**
