# Supplementary figures and images for: Therapeutic mechanism of baicalein in peritoneal dialysis-associated peritoneal fibrosis based on network pharmacology and experimental validation
Source: Front Pharmacol. 2023 May 17;14:1153503. doi: 10.3389/fphar.2023.1153503 (PMC10229821; doi:10.3389/fphar.2023.1153503)

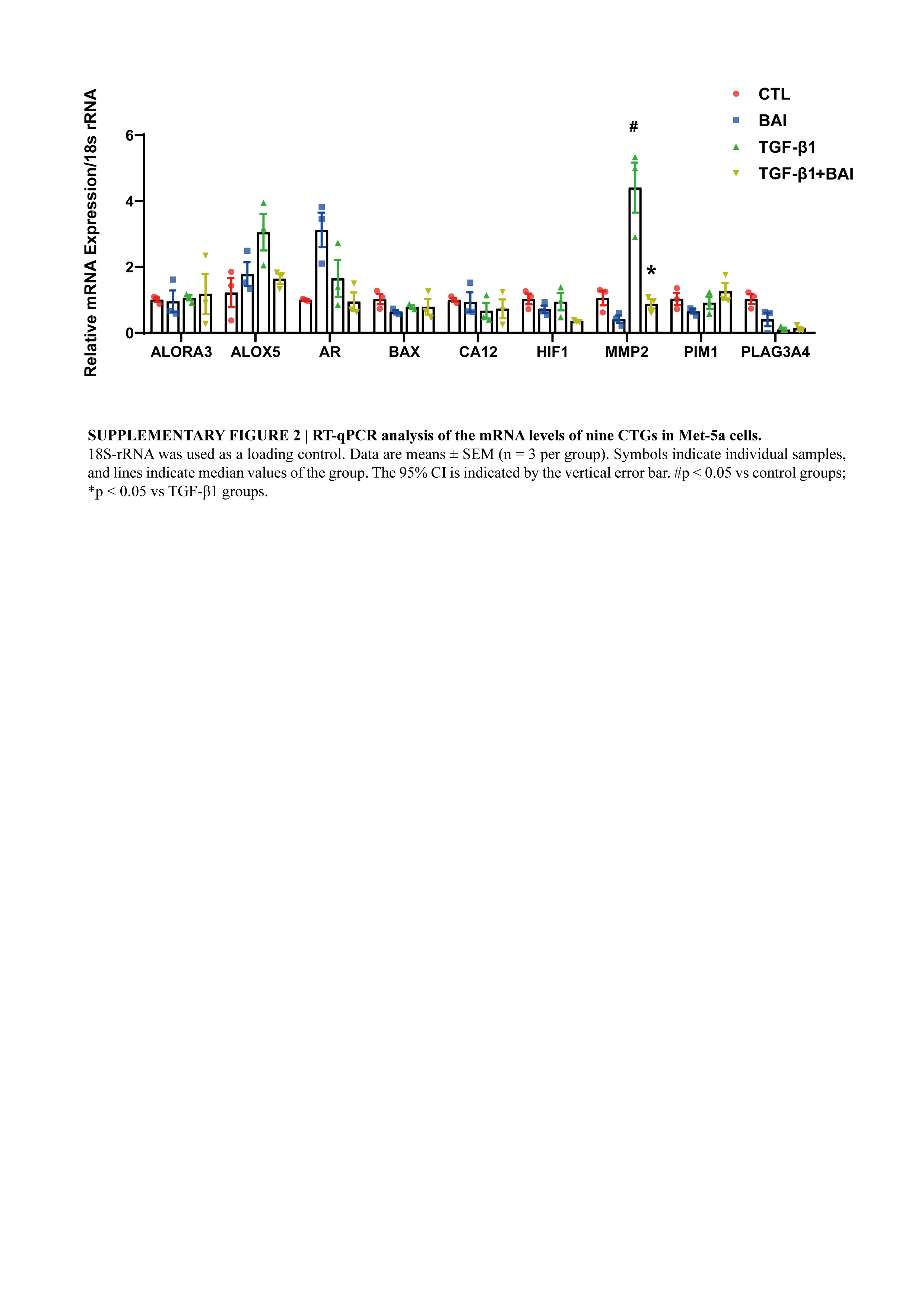

Supplement: Supplementary file 2 [file Image2.TIF]

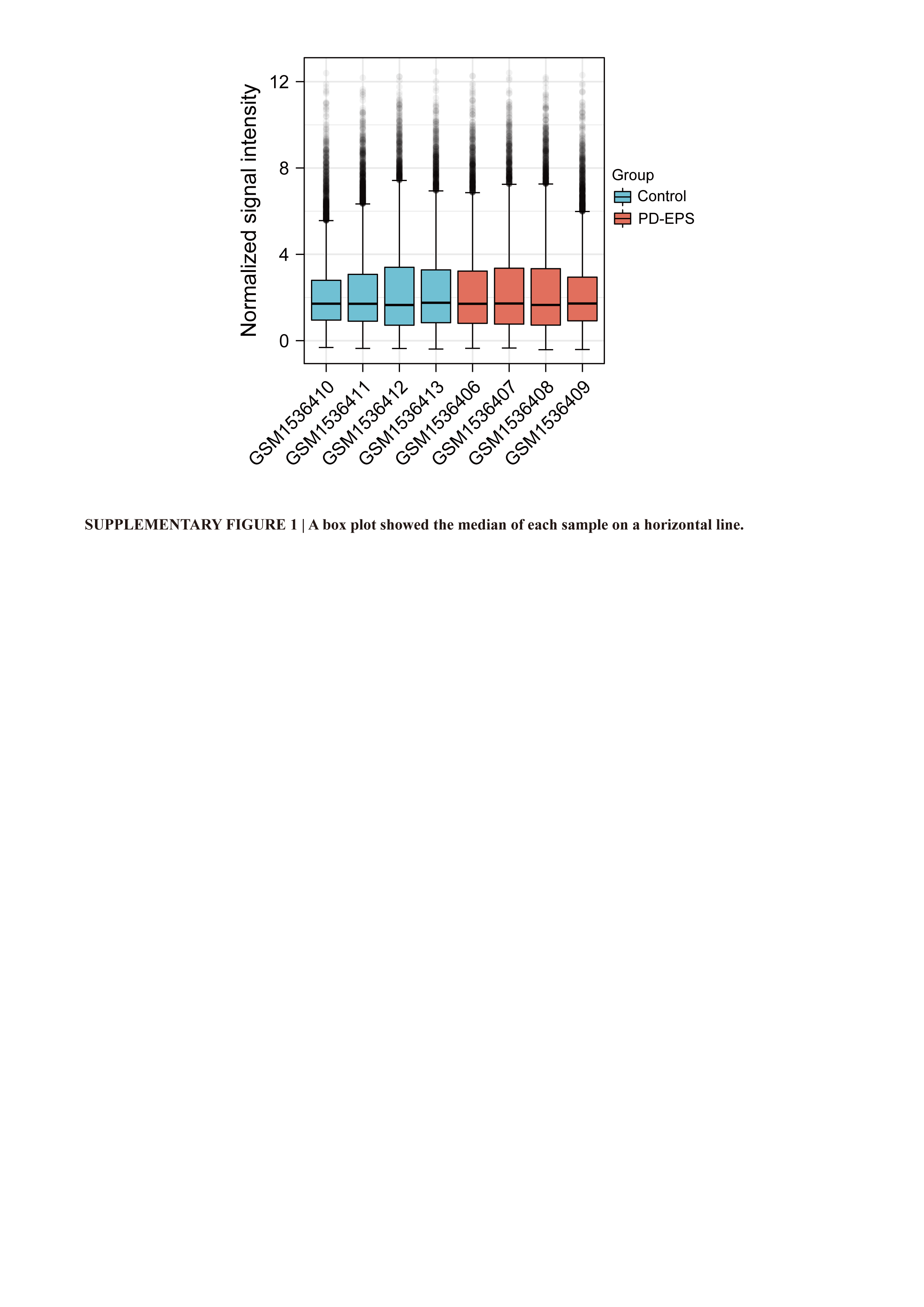

Supplement: Supplementary file 3 [file Image1.TIF]
